# A novel prognostic classification integrating lipid metabolism and immune co-related genes in acute myeloid leukemia

**DOI:** 10.3389/fimmu.2023.1290968

**Published:** 2023-11-10

**Authors:** Ding Li, Xuan Wu, Cheng Cheng, Jiaming Liang, Yinfeng Liang, Han Li, Xiaohan Guo, Ruchun Li, Wenzhou Zhang, Wenping Song

**Affiliations:** ^1^ Department of Pharmacy, The Affiliated Cancer Hospital of Zhengzhou University and Henan Cancer Hospital, Zhengzhou, China; ^2^ Henan Engineering Research Center for Tumor Precision Medicine and Comprehensive Evaluation, Henan Cancer Hospital, Zhengzhou, China; ^3^ Henan Provincial Key Laboratory of Anticancer Drug Research, Henan Cancer Hospital, Zhengzhou, China; ^4^ Academy of Medical Science, Zhengzhou University, Zhengzhou, China; ^5^ Department of Internal Medicine, Affiliated Cancer Hospital of Zhengzhou University, Henan Cancer Hospital, Zhengzhou, China; ^6^ Department of Medicine, The Second Affiliated Hospital of Guangzhou Medical University, Guangzhou, China

**Keywords:** acute myeloid leukemia, lipid metabolism, immunotherapy, drug sensitivity, prognostic signature

## Abstract

**Background:**

As a severe hematological malignancy in adults, acute myeloid leukemia (AML) is characterized by high heterogeneity and complexity. Emerging evidence highlights the importance of the tumor immune microenvironment and lipid metabolism in cancer progression. In this study, we comprehensively evaluated the expression profiles of genes related to lipid metabolism and immune modifications to develop a prognostic risk signature for AML.

**Methods:**

First, we extracted the mRNA expression profiles of bone marrow samples from an AML cohort from The Cancer Genome Atlas database and employed Cox regression analysis to select prognostic hub genes associated with lipid metabolism and immunity. We then constructed a prognostic signature with hub genes significantly related to survival and validated the stability and robustness of the prognostic signature using three external datasets. Gene Set Enrichment Analysis was implemented to explore the underlying biological pathways related to the risk signature. Finally, the correlation between signature, immunity, and drug sensitivity was explored.

**Results:**

Eight genes were identified from the analysis and verified in the clinical samples, including *APOBEC3C*, *MSMO1*, *ATP13A2*, *SMPDL3B*, *PLA2G4A*, *TNFSF15*, *IL2RA*, and *HGF*, to develop a risk-scoring model that effectively stratified patients with AML into low- and high-risk groups, demonstrating significant differences in survival time. The risk signature was negatively related to immune cell infiltration. Samples with AML in the low-risk group, as defined by the risk signature, were more likely to be responsive to immunotherapy, whereas those at high risk responded better to specific targeted drugs.

**Conclusions:**

This study reveals the significant role of lipid metabolism- and immune-related genes in prognosis and demonstrated the utility of these signature genes as reliable bioinformatic indicators for predicting survival in patients with AML. The risk-scoring model based on these prognostic signature genes holds promise as a valuable tool for individualized treatment decision-making, providing valuable insights for improving patient prognosis and treatment outcomes in AML.

## 1 Introduction

Acute myeloid leukemia (AML) is characterized by a clinically, epigenetically, and genetically heterogeneous disease with poor outcomes (1). Despite being initially sensitive to chemotherapy, most patients with AML ultimately experience relapse and die of progressive disease. Therefore, there is an urgent need for alternative treatment solutions. Advances in epigenomic and genomic characterization of AML have paved the way for the development and approval of novel targeted agents (2). Immunotherapy is also a promising strategy for long-term disease control. However, acquired resistance to targeted agents and a low response to immunotherapy still cause treatment failure (3). Thus, novel therapeutic targets and prognostic biomarkers are urgently required to guide clinical practice and predict the survival of patients with AML.

Emerging evidence suggests that metabolic disruptions, particularly those involving certain metabolites and associated pathways, are crucial factors in the development and progression of leukemia. Lipids and their derivatives play critical roles in energy generation and form the structural basis of cellular and organelle membranes. Extensive research conducted over numerous years has explored the impact of lipid metabolism on AML, leading to recent breakthroughs (4). As a lipid category, fatty acids represent an appealing therapeutic target that supports increased biomass, membrane biogenesis, energy production, and lipoprotein generation in dividing AML cells (5). AML is associated with the overexpression and constant activation of sphingosine kinase 1, an enzyme responsible for producing sphingosine 1-phosphate from sphingosine. Remarkably, the inhibition of sphingosine kinase 1 induces apoptosis in AML blasts and leukemic stem cells obtained from patients (6, 7). Consequently, control of lipid metabolism reprogramming has emerged as a promising therapeutic target for enhancing the prognosis of individuals diagnosed with AML. Therefore, we previously constructed a prognostic signature with high specificity and sensitivity for estimating the prognosis of AML patients based on lipid metabolism-related genes (LMRGs) (8). The findings showed that the risk signature had remarkable specificity and sensitivity in estimating the outcomes of AML patients. And, consistent with the findings of other studies, interventions aimed at modulating lipid metabolism have the potential to impact not only tumor cells, but also immune cells (9, 10). We found that the lipid metabolism-related risk signature was closely associated with the immune tumor microenvironment (TME) and response to immunotherapy in AML.

As is same to solid tumor cells, AML cells are capable of developing an immunosuppressive microenvironment in which both adaptive and innate immune responses are profoundly disrupted (11, 12). Emerging evidence indicates that lipids are crucial for driving this dysregulated state. In acidic, hypoxic, and nutrition-deficient TMEs, both the cancer and immune cells tend to depend on the lipids for energy storage, building cellular membranes, and generating signaling molecules. Consequently, the dysregulation of lipid metabolism within the TME can have a profound impact on tumorigenesis, subsequent progression, and metastasis. Within this complex TME, lipids act as double-edged swords capable of either supporting antitumor or promoting pro-tumor immune responses (9, 12). These contradictory results present a dilemma, as simply inhibiting or stimulating a single lipid metabolic pathway within the TME fails to achieve optimal results. The models constructed with a single feature exhibited relatively weaker validity and robustness than those constructed with multiple features. Therefore, there is an urgent need for a comprehensive understanding of a multi-featured signature model specifically tailored for patients with AML, along with an exploration of its prognostic implications.

In this study, we integrated genes related to immunity and lipid metabolism to develop a prognostic signature based on the interactions between antitumor immunity and lipid metabolism.

## 2 Materials and methods

### 2.1 Data collection and preparation

The clinical data and RNA-sequencing profile of the patients with AML (**Supplementary Table 1**) came from The Cancer Genome Atlas (TCGA) database (https://www.cancer.gov/tcga/). Prior to analysis, all transcriptome data for fragments per kilobase of transcript per million mapped reads were log-transformed and subsequently converted to transcripts per million. Baseline features of the AML patients involved in the risk signature are displayed in **Supplementary Table 2**.

For external validation, three independent datasets (GSE12417, GSE37642, and GSE71014) along with the clinical data were acquired from the GEO database, available at https://www.ncbi.nlm.nih.gov/geo/.

### 2.2 Identification of immune- and lipid metabolism-related prognostic genes

Here, we incorporated a comprehensive approach to identify genes associated with lipid metabolism. Specifically, we included all genes from 34 LMRG sets sourced from the Molecular Signature Database (MsigDB; available at https://www.gsea-msigdb.org/gsea/msigdb/) (13). By considering the intersection of these gene sets, we derived a final set of 1,996 LMRGs. For detailed information regarding the LMRG sets, please refer to **Supplementary Table 3**. A collection of 1,793 immune-related genes was acquired from the ImmPort database, available at https://www.immport.org/ (14). Details of the immune-related genes (IRGs) are displayed in **Supplementary Table 4**. The integration of LMRGs and IRGs was performed to conduct a prognostic analysis of AML, and 180 prognostic genes (p <0.01) were acquired for the subsequent analyses.

### 2.3 Development and validation of a prognostic lipid metabolism and immune co-related signature

A total of 144 samples from the AML cohort in the TCGA database were then randomly divided into the training (N = 72) and validation (N = 72) datasets in a 1:1 ratio. First, we used univariate Cox regression to identify LMRGs and IRGs with prognostic role in the training dataset. Then, least absolute shrinkage and selection operator (LASSO) Cox regression analysis with the R package (version 3.6.1) “glmnet,” a novel risk-scoring model with eight genes was developed as follows:

Risk score = expAPOBEC3C × 0.188873061 + expMSMO1 × 0.176721847 + expATP13A2 × 0.096045519 + expSMPDL3B × 0.077828708 + expPLA2G4A × 0.071836509 + expTNFSF15 × 0.027983123 + expIL2RA × 0.022815855 – expHGF × 0.044508523

Subsequently, patients with AML in the training dataset were classified into low-risk group and the high-risk group by the median cutoff risk score. The Kaplan-Meier survival curve was performed to compare the differences between the two risk groups. The receiver operating characteristic (ROC) curves were constructed to assess the validity of the risk signature.

The validity of the risk signature was verified using samples from the GSE12417, GSE37642, and GSE71014 cohorts. The same analyses used for the training dataset were used to calculate the risk scores of samples from the GEO cohorts.

### 2.4 Clinical correlation and subgroup analyses

To assess the clinical significance and prognostic utility of the risk signature, we extracted the clinical data of 144 patients with AML in the TCGA database, and these variables included age (**>=** 60 years or < 60 years), gender (female or male), chromosome status (normal or abnormal), and gene mutation (FLT3, NPM1, RAS, and IDH1 mutation or not) (**Supplementary Table 5**). Then, Kaplan–Meier curves were initially generated to explore the prognostic role of each gene included in the risk signature (15).

### 2.5 Functional enrichment analysis

The TCGA database contained genomic data from 144 samples in the AML cohort, which were classified into either high-risk or low-risk groups based on their risk score. Using the GSEA v4.1.0 software (https://www.gsea-msigdb.org/gsea/index.jsp), the hallmark gene set (h.all.v7.2.symbols.gmt) was employed for enrichment analysis, with the phenotypic label being the high-risk group versus the low-risk group. The number of permutations used was 1000, while all other settings were set to default values (13). Statistically significant findings were defined as p <0.05 and q <0.05.

### 2.6 Nomogram construction and assessment

By integrating the risk scores and clinical data of 144 patients with AML in the TCGA database, we constructed nomogram survival models for overall survival (OS) by the “rms” R package, incorporating both univariate and multivariate results. The calibration curve estimate was then adjusted for optimism by using a bootstrap procedure (16). In addition, ROC curves were generated to validate the predictive capacity of the risk signature with clinical characteristics.

A total of 144 patients with AML in the TCGA database were classified into low-risk group and the high-risk group by the median cutoff risk score. The CIBERSORT algorithm was performed to estimate the infiltration levels of various immune cell types (17). Tumor immune dysfunction and exclusion (TIDE) data for AML was acquired from http://tide.dfci.harvard.edu/. The TIDE algorithm was developed to generate TIDE scores and to accurately evaluate the response of immunotherapy agents in patients with cancer (18). Lower TIDE scores indicate better outcomes. The immunotherapy response of each patient was evaluated by the gene expression profiles.

### 2.8 Pharmaceutical screening

A total of 144 patients with AML in the TCGA database were classified into low-risk group and the high-risk group by the median cutoff risk score. Then, we employed the “pRRophetic” R package in the Genomics of Drug Sensitivity in Cancer (GDSC) database to determine the varying susceptibilities to the drug between high- and low-risk groups. The half maximal inhibitory concentration (IC_50_) value, which indicates the concentration at which cell growth is inhibited by 50%, was used as a metric of drug sensitivity (19, 20). Stringent filtration conditions (p <0.01) were used.

### 2.9 Quantitative real-time PCR

Details of the PCR operation was carried out in accordance with previous study (21). Samples of health donor and patients with AML were collected from Henan Cancer Hospital and approved by Medical Ethics Committee of The Affiliated Cancer Hospital of Zhengzhou University (approval no. 2023-KY-0104-001). The PCR primers were purchased from SangonBiotech (Sangon, Zhengzhou, China). And, the primer sequences in this study were showed in the **Supplementary Table 6**.

## 3 Results

### 3.1 Construction of an eight-gene signature with high accuracy of prognosis prediction

**Figure 1:**
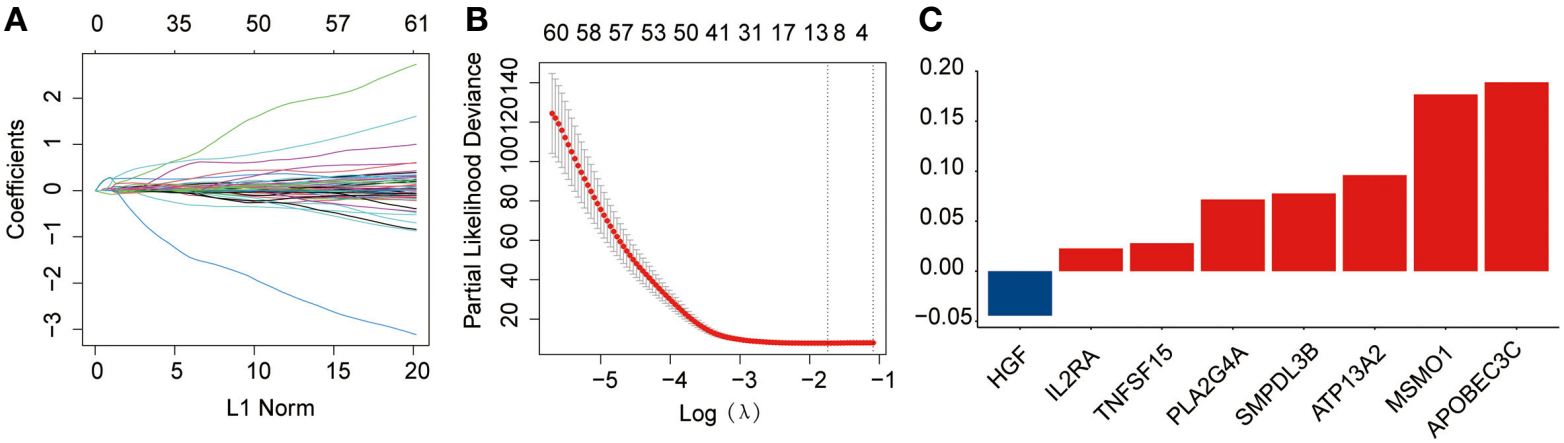
Development of the prognostic risk signature in the training dataset. **(A)** The least absolute shrinkage and selection operator (LASSO) model was subjected to ten fold cross-validation for variable selection. **(B)** LASSO coefficient profile of identified crucial genes. **(C)** Coefficient profile of the eight prognostic genes.

Briefly, 1,996 LMRGs and 1,793 IRGs in AML were included, of which 180 candidate prognostic genes were subsequently identified using univariate Cox regression analysis (**Figure 1A**). LASSO Cox regression analysis finally identified eight crucial genes for lipid metabolism- and immune-related prognostic signatures according to the optimal λ value (**Figures 1B, C**). Among them, there were five LMRGs (*MSMO1*, *ATP13A2*, *SMPDL3B*, *PLA2G4A*, and *TNFSF15*) and three IRGs (*APOBEC3C*, *IL2RA*, and *HGF*). Except for *HGF*, all other seven signature genes are detrimental factors with a hazard ratio (HR) >1. The risk score for each AML sample in this study was calculated by the formula described in Section 2.3.

**Figure 2:**
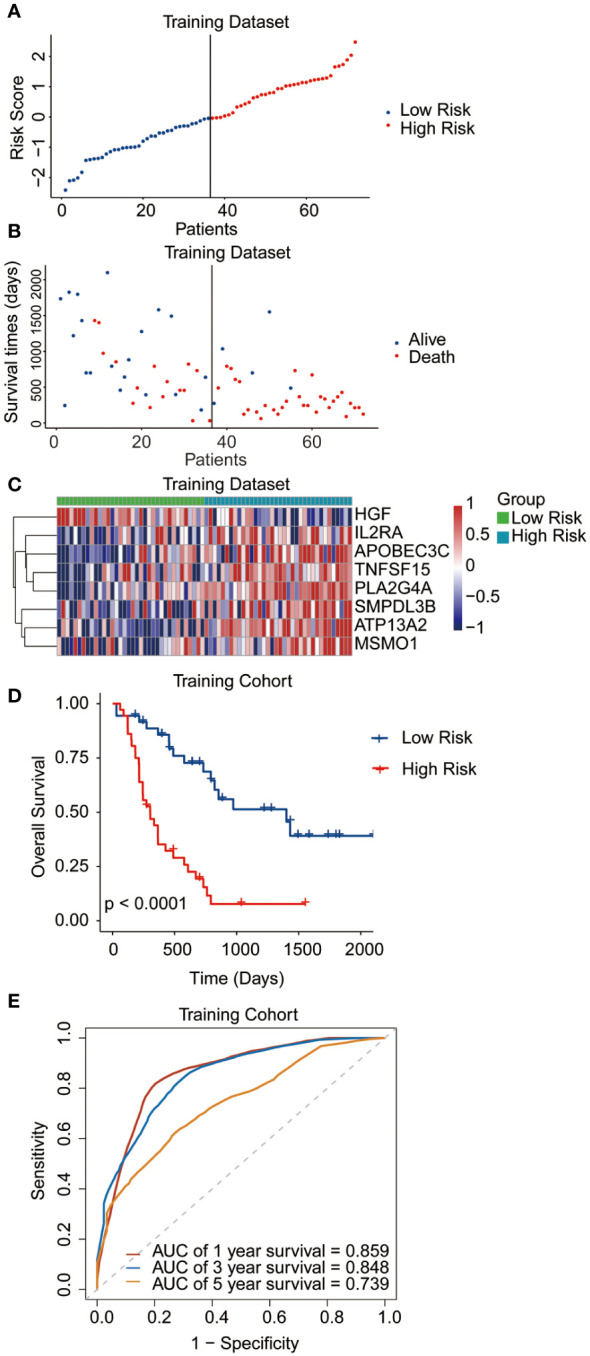
Performance of the prognostic signature in the training dataset. **(A)** The risk curve of each AML sample was defined by risk score. **(B)** Scatter plots showing the survival status of each sample. **(C)** Heat map of the expression of the eight selected genes. **(D)** Kaplan-Meier survival curves between the two risk groups. **(E)** The receiver operating characteristic (ROC) curves for overall survival at 1, 3, and 5 years.

**Figure 3:**
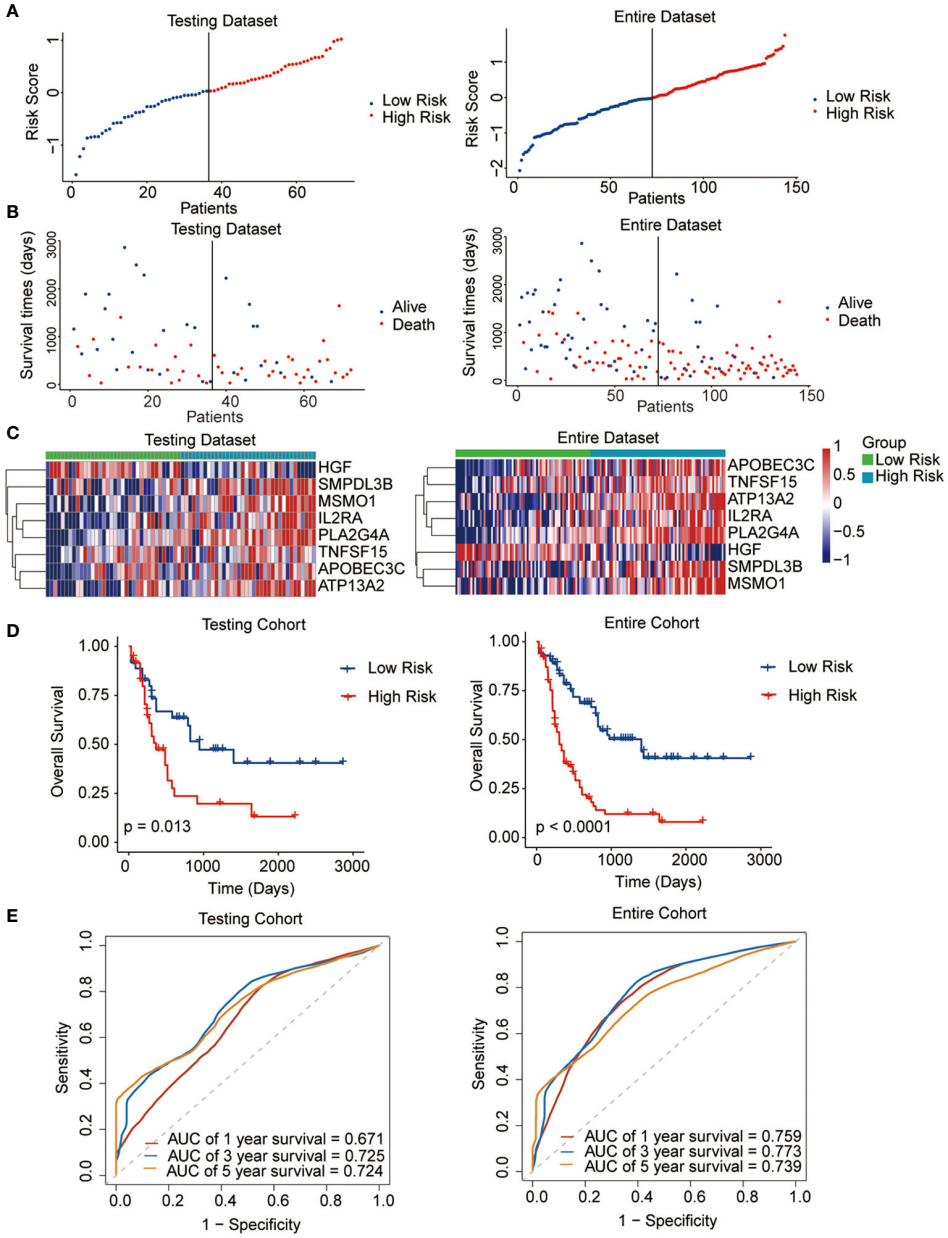
Performance of the prognostic signature in the testing and entire datasets. **(A)** The risk curve of each AML sample was defined by risk score. **(B)** Scatter plots showing the survival status of each sample. **(C)** Heat map of the expression of the eight selected genes. **(D)** Kaplan-Meier survival curves between the two risk groups. **(E)** The receiver operating characteristic (ROC) curves for overall survival at 1, 3, and 5 years.

The median risk score was regarded as the cut-off value to classify the training TCGA cohort into the high-risk and low-risk groups (**Figure 2A**). The scatter plot indicated that high-risk patients were significantly associated with a high mortality rate compared to that of low-risk patients (**Figure 2B**). The gene expression heatmap illustrates that, except for *HGF*, all other seven signature genes were upregulated in the high-risk group (**Figure 2C**). Kaplan-Meier curve analysis demonstrated that high-risk patients suffered significantly worse survival outcomes than low-risk ones (**Figure 2D**). The AUC reached 0.807, 0.848, and 0.843 at 1, 3, and 5 years, respectively (**Figure 2E**). In addition, results for the testing and entire datasets were consistent with those from the training dataset (**Figures 3A–E**). The above results demonstrated that the potential prognostic signature showed great specificity and sensitivity in estimating the prognosis of AML patients.

### 3.2 External validation of the risk signature in the GEO cohorts

**Figure 4:**
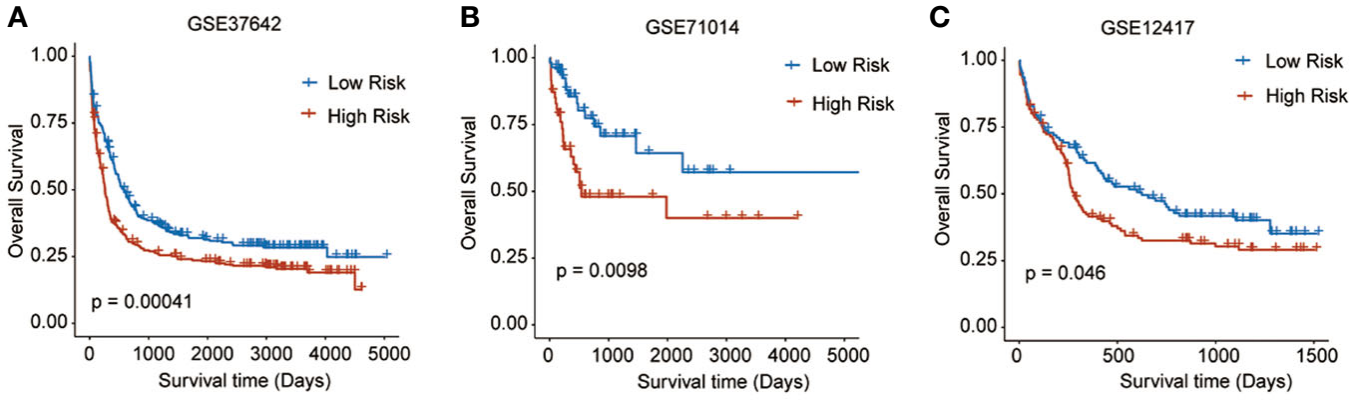
Survival analyses performed on all three GEO validation datasets. **(A)** GSE37642: p = 0.00041, **(B)** GSE71014: p = 0.0098, **(C)** GSE12417: p = 0.046).

To validate the predictive reliability of this prognostic signature, we screened and included three GEO datasets as external validation cohorts. After calculating the risk scores for each sample in these datasets, we assigned patients to high- and low-risk groups by the median cut-off value of these scores. Survival analyses performed on all three validation datasets consistently demonstrated that in the high-risk patients with AML experienced significantly worse OS outcomes than the low-risk ones (GSE37642, p = 0.00041; GSE71014, p = 0.0098; GSE12417, p = 0.046) (**Figures 4A–C**).

### 3.3 Correlation between the clinical characteristics and prognostic signature

**Figure 5:**
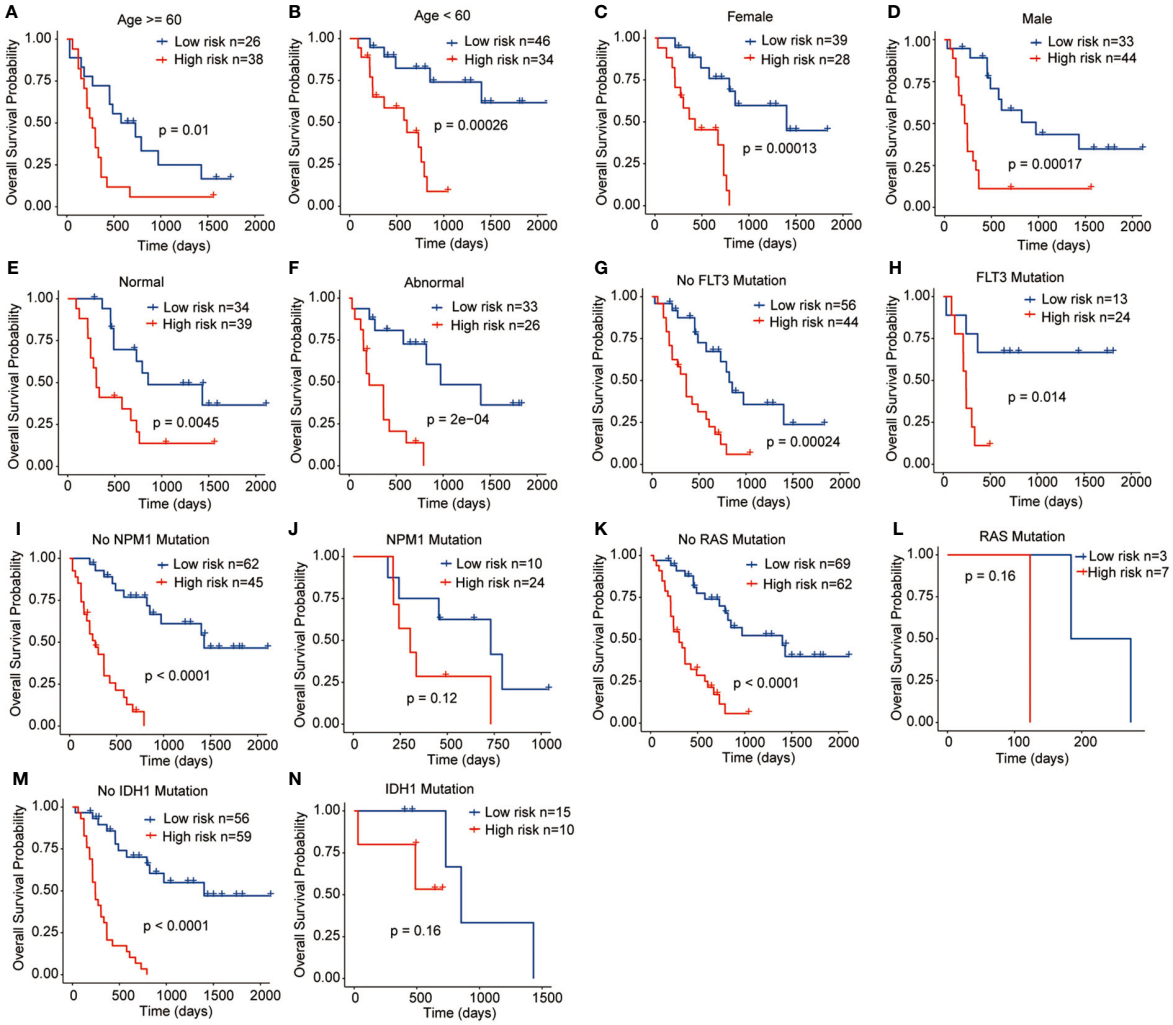
Relationships between the prognostic signature and clinicopathological characteristics. **(A)** Age **>=** 60 years, **(B)** Age < 60 years, **(C)** Female, **(D)** Male, **(E)** Normal chromosome, **(F)** Abnormal chromosome, **(G)** No FLT3 mutation, **(H)** FLT3 mutation, **(I)** No NPM1 mutation, **(J)** NPM1 mutation, **(K)** No RAS mutation, **(L)** RAS mutation, **(M)** IDH1 mutation, **(N)** IDH1 mutation.

To assess the clinical significance and prognostic utility of the risk signature, Kaplan-Meier curves were initially generated to explore the prognostic role of each gene included in the risk signature. These variables included age (>= 60 years or < 60 years), gender (female or male), chromosome status (normal or abnormal), and gene mutation status (FLT3, NPM1, RAS, and IDH1 mutation or not). The results revealed that regardless of the clinicopathological features, high-risk patients tend to have the worst OS outcomes, indicating the stable performance of the prognostic risk signature (**Figures 5A–N**).

### 3.4 Nomogram analysis

**Figure 6:**
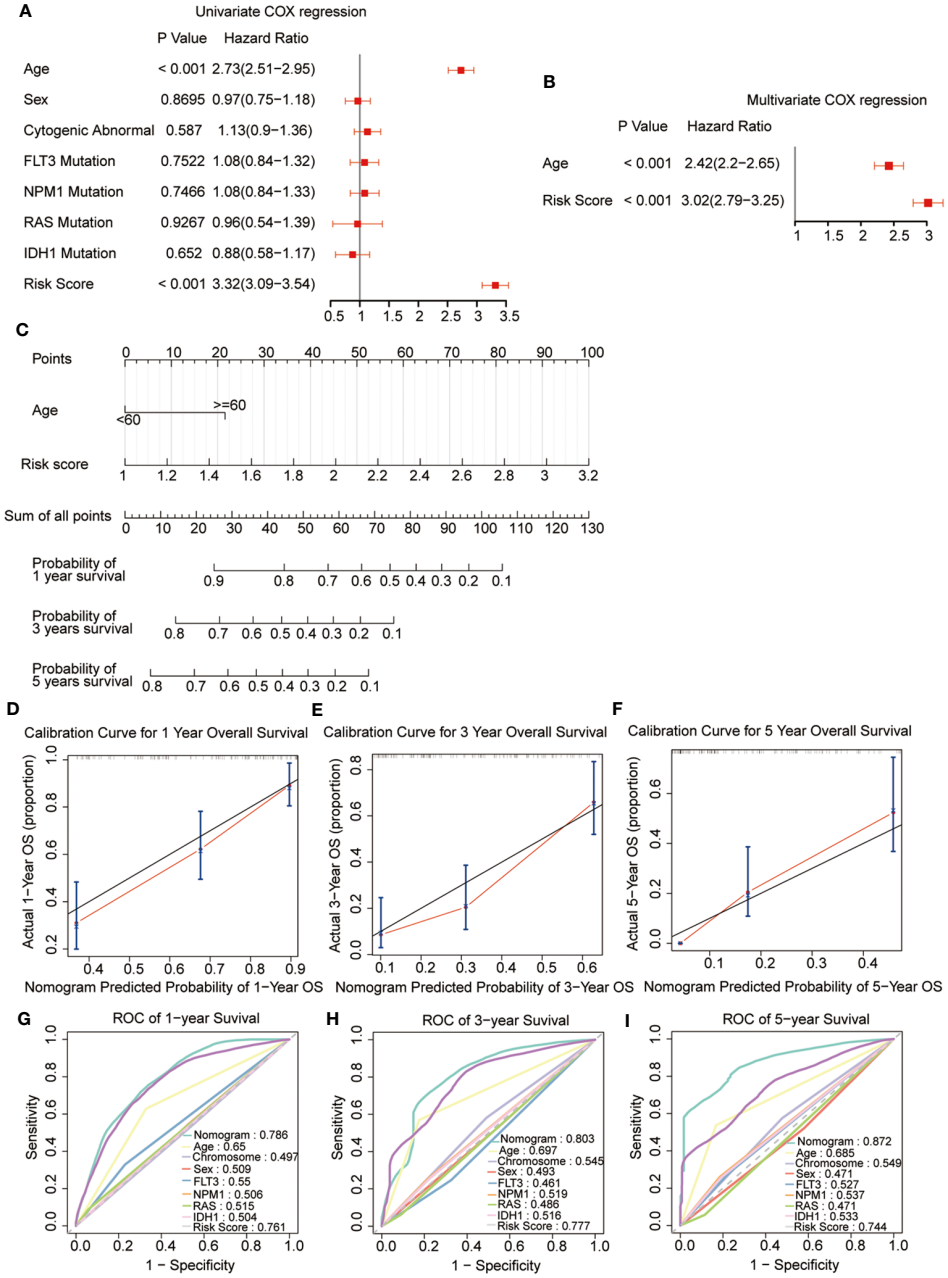
Construction and validation of the nomogram. **(A, B)** Univariate and multivariate Cox regression of the prognostic signature and clinical characteristics. **(C)** The developed nomogram to estimate the survival possibilities of patients with AML. **(D-F)** Calibration blots of the agreement between the predicted overall survival and observed overall survival at 1, 3, and 5 years. **(G–I)** The ROC curves for overall survival at 1, 3, and 5 years.

Univariate combined with multivariate Cox regression analyses were preformed to explore whether the risk signature and clinicopathological parameters, including age, sex, chromosomal status, and gene mutations, were the independent prognostic factors. The results showed that the risk scores (HR = 3.02; 95% CI 2.79-3.25) and age (HR = 2.42; 95% CI 2.2-2.65) were the independent prognostic factors for survival (**Figures 6A, B**). In addition, a nomogram was developed using age and risk scores to accurately predict the survival rates at 1-, 3-, and 5-year in patients with AML, which suggested that a higher total score suggested worse survival. The result showed that the prognostic signature had the greatest impact on OS (**Figure 6C**). Meanwhile, the calibration curve demonstrated a strong agreement between the predicted and observed OS at 1-, 3-, and 5-year intervals, indicating the excellent predictive accuracy of the prognostic signature (**Figures 6D–F**). Furthermore, the 1-, 3-, and 5-year survival ROC analyses showed that the AUCs for the nomogram and risk scores were superior to the other variables, such as age, chromosomal status, sex, as well as FLT3, NPM1, RAS, and IDH1 mutations (**Figures 6G–I**). These results showed that the nomogram and risk score provided a higher practical value for prognostic prediction than the other variables.

### 3.5 Biological functions and pathway analysis

**Figure 7:**
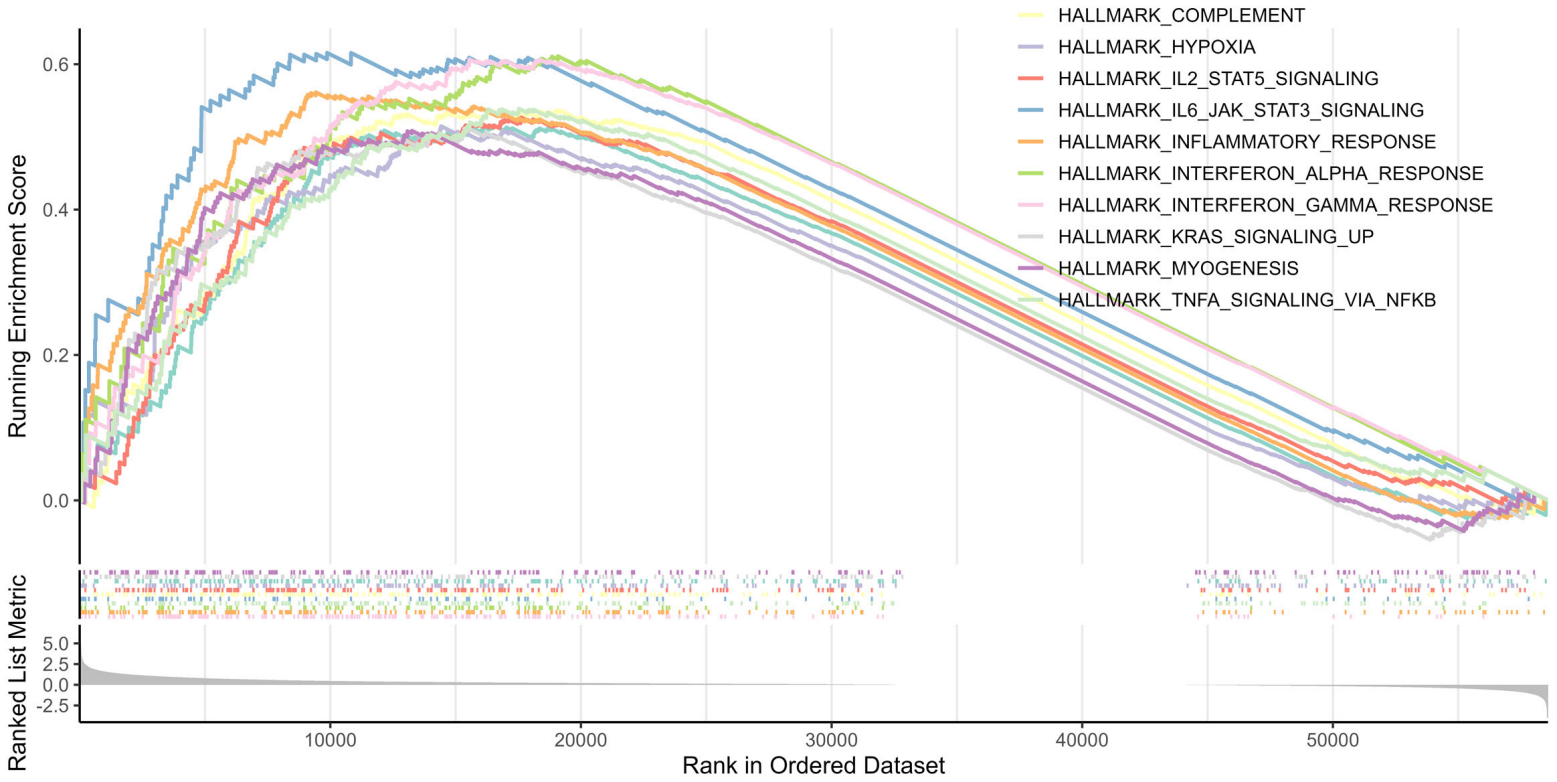
Top 10 significantly enriched pathways in the GSEA.

GSEA was performed between the two risk groups to identify the underlying biological functions and pathways associated with the risk score. The results indicated that interferon γ, inflammatory, and interferon α responses, as well as TNFα signaling via NF-κB, complement, IL2-STAT5 signaling, IL6-JAK-STAT3 signaling, allograft rejection, hypoxia, and KRAS signaling pathway were enriched, which are central in mediating host responses to inflammation and antitumor immunity (**Figure 7**).

### 3.6 Correlation between the prognostic signature and tumor immune microenvironment

**Figure 8:**
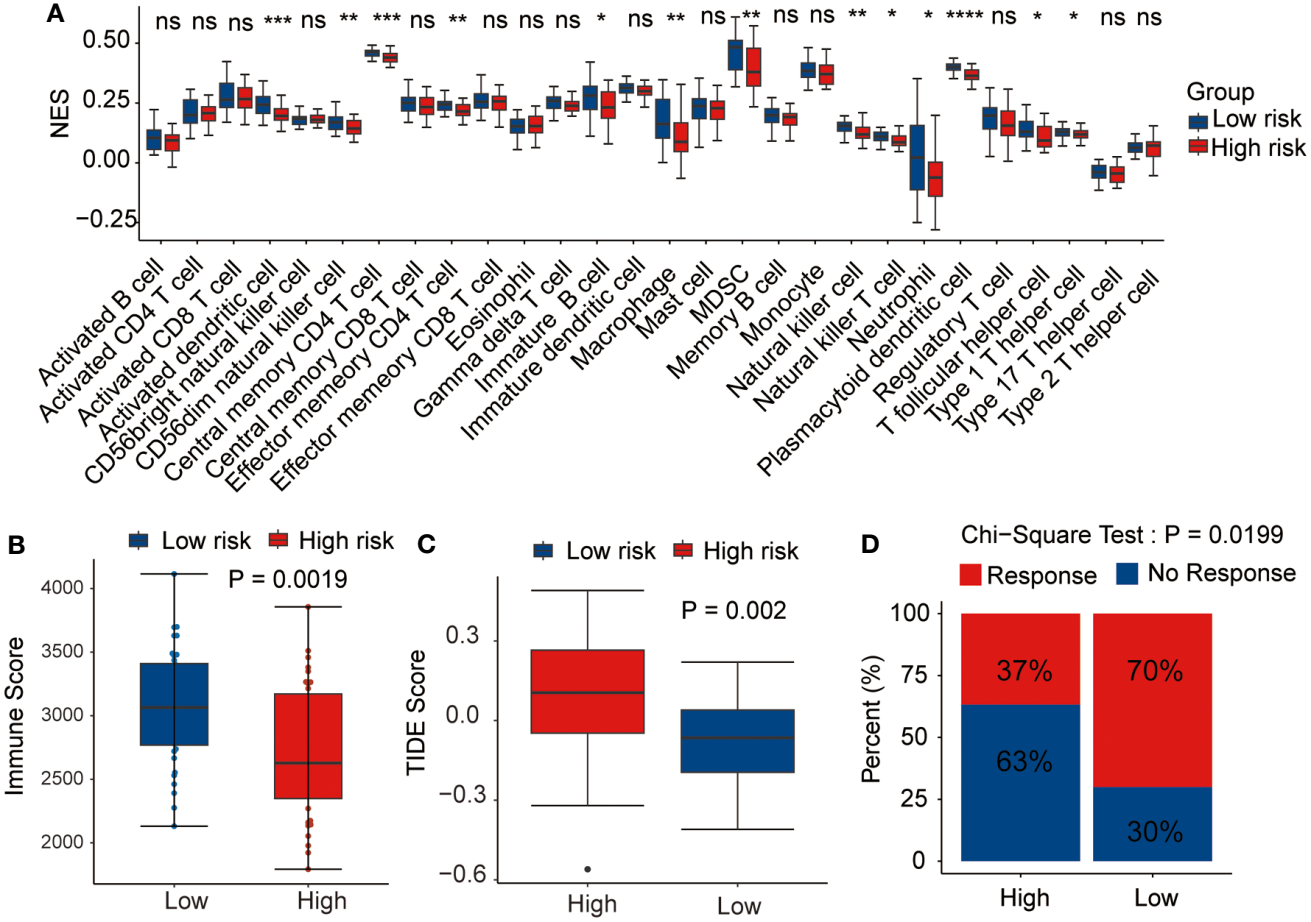
Relationship between the prognostic signature and tumor microenvironment. Correlation of the risk score with **(A)** immune infiltration level, **(B)** immune score, **(C)** tumor immune dysfunction and exclusion (TIDE) score, and **(D)** immunotherapy response. *p <0.05, **p <0.01, ***p <0.001, ****p <0.0001, ns, not statistically significant.

As the antitumor immunity-related signaling pathways were significantly enriched in the GSEA analysis, we evaluated the correlation of the prognostic risk signature with immune state in each patient with AML. CIBERSORT algorithm was performed to estimate the infiltration levels of various immune cell types in the TME. The results demonstrated that high-risk patients had a lower fraction of activated dendritic cells, CD56dim NK cells, effector memory CD4 T cells, macrophages, immature B cells, MDSCs, NK cells, NK T cells, neutrophils, T follicular helper cells, plasmacytoid dendritic cells, and type 1 T helper cells (**Figure 8A**). Then, the immune scores and the TIDE scores of each sample were calculated, and the results demonstrated that the high-risk samples hold lower immune scores and higher TIDE scores than the low-risk samples (**Figures 8B, C**), indicating that high-risk patients were associated with enhanced tumor immune escape ability. Moreover, we assessed the disparity in the response rates to immunotherapy between the two risk groups. Notably, the samples from the low-risk group exhibited higher immunotherapy response rates than those from the high-risk group (**Figure 8D**). Based on these outcomes, we ascertained that the risk signature could indicate the immune cell infiltration and the response to immunotherapy in AML.

### 3.7 Drug sensitivity analysis

**Figure 9:**
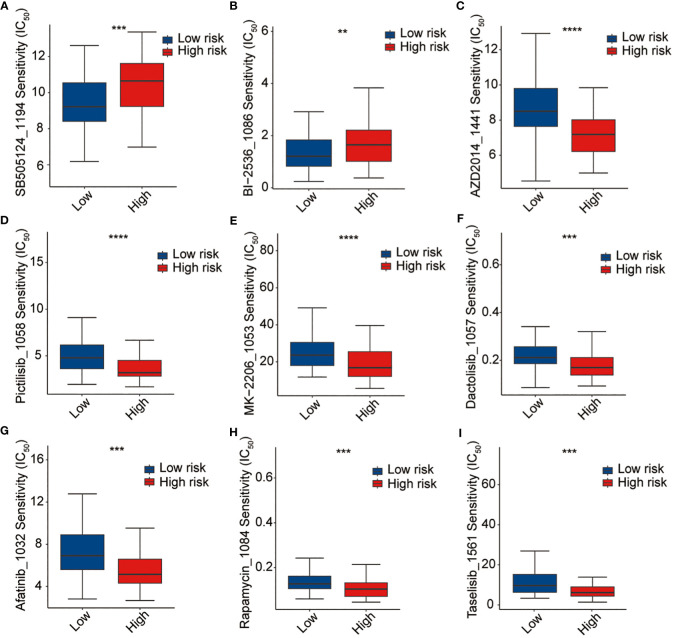
Drug sensitivity analysis. **(A)** SB-505124, **(B)** BI2536, **(C)** AZD2014, **(D)** pictilisib, **(E)** MK-2206, **(F)** dactolisib, **(G)** afatinib, **(H)** rapamycin, and **(I)** traselisib. **p <0.01, ***p <0.001, ****p <0.0001.

Thereafter, the pRRophetic package were used to further analyze the sensitivity of antitumor drugs based on the IC_50_ available in the GDSC database for patients with AML (19, 20). In our study, we successfully identified a total of 198 small molecular compounds that exhibited significantly diverse responses between the high-risk and low-risk groups (**Supplementary Table 7**). The results showed that the high-risk group showed a lower sensitivity to BI2536 (PLK1 inhibitor) and SB-505124 (TGFβR inhibitor), whereas they were sensitive to several other drugs such as AZD2014 (mTOR inhibitor), pictilisib (PI3Kα/δ inhibitor), MK-2206 (Akt inhibitor), dactolisib (dual pan-class I PI3K and mTOR kinase inhibitor), afatinib (EGFR inhibitor), rapamycin (FRAP/mTOR inhibitor), and taselisib (PI3K inhibitor targets PIK3CA mutations), even though none of these is currently used in the treatment of AML (**Figure 9**). The outcomes of our study offer promising molecular candidates for targeted therapy that can be utilized in the treatment of AML patients.

## 4 Discussion

Here, we studied the role of LMRGs and IRGs in the prognosis of patients with AML. By analyzing large-scale genomic and clinical datasets from TCGA and GEO databases, we identified an eight-gene signature that demonstrated robust prognostic value and potential clinical applications in AML. We performed additional analysis on the expression of eight signature genes in the high and low-risk groups across multiple cohorts, including TCGA, GSE12417, GSE37642, and GSE71014. The findings demonstrated that MSMO1, ATP13A2, SMPDL3B, PLA2G4A, TNFSF15, APOBEC3C, and IL2RA were upregulated in the high-risk group, whereas HGF was downregulated. Survival analysis indicated that patients with high expression of these signature genes, except for HGF, experienced worse OS outcomes. These results provide further evidence that these genes may function as detrimental factors, while HGF may serve as a protective factor (**Supplementary Figures 1** and **2**). The relative expression of these eight signature genes were also detected in the clinical samples (**Supplementary Figure 3**).

*APOBEC3C* is a member of the APOBEC family that plays important but distinct roles in host defense and mediates C-to-T mutagenesis in cancers. A previous study indicated a negative correlation between *APOBEC3C* mRNA expression and base substitution mutations in estrogen receptor-negative breast cancer (22). Qian et al. found that *APOBEC3C* was significantly upregulated in pancreatic ductal adenocarcinoma compared with that in normal pancreatic tissues and predicted worse survival rates (23). Jiang et al. found that increased *APOBEC3C* expression was related to hematopoietic stem and progenitor cell proliferation and an increased C-to-T mutational burden during disease progression in patients with myeloproliferative neoplasm (24).

Methylsterol monooxygenase 1 (MSMO1), an intermediate enzyme involved in cholesterol and fatty acid biosynthesis, acts as a novel mediator of chemoresistance in cancer (25). A previous study revealed that MSMO1 plays crucial roles in tumorigenesis and progression and is a promising prognostic biomarker for cervical squamous cell carcinoma (26).

ATPase cation transporting 13A2 (ATP13A2/PARK9), a late endolysosomal transporter, regulates membrane association, cellular α-synuclein multimerization, and externalization and is genetically implicated in neurodegenerative disorders (27). Zhang et al. revealed that ATP13A2 activates the pentose phosphate pathway via the TFEB-PGD axis to facilitate colorectal cancer growth (28).

As the negative regulator of Toll-like receptor signaling, Sphingomyelin Phosphodiesterase Acid Like 3B (SMPDL3B) plays a crucial role in innate immunity and at the interface of membrane biology. Qu et al. demonstrated that SMPDL3B expression indicates poor prognosis and contributes to AML progression (29).

The cytosolic phospholipase, PLA2G4A, is crucial for the pathogenesis of FLT3-ITD-mutated AML (30). Higher PLA2G4A expression results in worse OS and mutations in NRAS, which are known to contribute to the development of myelodysplastic syndrome development (31).

Tumor necrosis family superfamily member 15 (TNFSF15) promotes lymphatic metastasis by upregulating vascular endothelial growth factor-C in a lung cancer mouse model (32). Lu et al. showed that increased TNFSF15 expression indicates worse prognosis in oral cancer (33).

Excessive expression of *IL2RA*, the gene encoding the alpha chain of the interleukin-2 receptor, has been linked to chemotherapy resistance and unfavorable outcomes in AML (34). *IL2RA* enhances cell proliferation and cell cycle activity while suppressing apoptosis in both human AML cell lines and primary cells. In two genetically modified mouse models of AML, *IL2RA* hampered cell differentiation, facilitated stem cell-like characteristics, and was essential for leukemia development. Antibodies targeting *IL2RA* have demonstrated the ability to inhibit leukemic cells without affecting normal hematopoietic cells, and their combined effects with other anti-leukemic agents have shown potential synergy. Consequently, *IL2RA* is a promising therapeutic target in AML because it regulates key processes, such as proliferation, differentiation, apoptosis, stem cell-related properties, and leukemogenesis (35).

As a multifunctional cytokine, hepatocyte growth factor (HGF) regulates cell growth, movement, and tissue regeneration in various epithelial cells (36). HGF binds to its receptor c-Met and activates its kinase activity, initiating signaling pathways such as JAK/STAT3, PI3K/Akt/NF-κB, and Ras/Raf. Aberrations in the HGF/MET pathway act as diagnostic, predictive, and prognostic biomarkers for cancers (37). HGF has been discovered to regulate the activity of various immune cell types, including B cells, T cells, and natural killer cells, which are important components of the anti-tumor immune response. By enhancing the immune surveillance and anti-tumor effects, HGF may contribute to reducing the risk of AML development or progression. While, it’s worth noting that the exact mechanisms by which HGF influences AML risk are still being investigated, and further studies are required to fully reveal its role in the disease. Nonetheless, the association between HGF and a reduced risk in AML highlights the potential importance of this growth factor in the development and treatment of the disease.

The risk score defined by the prognostic signature defined in this study effectively stratified patients with AML into low- and high-risk groups with significantly different survival outcomes. These results are consistent with those of the external validation cohorts from the GEO dataset. Regardless of age, sex, cytogenetic abnormalities, or gene mutations, patients in the high-risk group consistently exhibited worse OS outcomes, further supporting the reliability and generalizability of the prognostic risk signature.

To enhance the clinical utility of our findings, we constructed nomograms that integrated the risk scores derived from the eight-gene signature with other clinical factors. The ROC and calibration curves further confirmed the higher predictive accuracy of the prognostic signature and nomograms compared with the clinical variables, such as age, sex, cytogenetic abnormalities, and gene mutations, indicating their potential as reliable tools for personalized treatment decision-making.

GSEA between the two risk groups sheds light on the underlying biological mechanisms associated with the prognostic signature. Many antitumor immunity-related pathways were enriched, suggesting the involvement of immune dysregulation in AML prognosis. This could lead to the distinction in the immunotherapy response against cancer and the treatment response between the two risk groups.

Then, the correlation between the immune cell infiltration and risk score was explored. The low-risk group showed higher proportions of effector memory CD4 T cells, macrophages, NK cells, NK T cells, T follicular helper cells, Type 1 T helper cells, and other immune cell subtypes. The negative correlation between the immune cell infiltration and risk score suggests that patients in the high-risk group may have impaired immune status. The immune and immune escape scores were then calculated, and the results demonstrated a poorer immune state and stronger immune escape ability in the high-risk group, which may affect the response to immunotherapy. Furthermore, in the high-risk group, there was a notable decrease in the expression level of common immune checkpoints such as PD1, PDL1, PDL2, and CTLA4 (**Supplementary Figure 4**). These findings indicate that the identified signature holds promise as a valuable tool for assessing the effectiveness of immunotherapy in individuals with AML. Additionally, our prediction results of the immunotherapy response rate further verified this conclusion, which showed that low-risk patients had higher immunotherapy response rates than that of high-risk patients. This finding highlights the potential importance of immune modulation in AML treatment. Future research could focus on understanding the underlying mechanisms that contribute to immune suppression in high-risk patients and explore strategies to enhance immune cell function in these individuals.

In line with the potential impact on the immunotherapy response, we evaluated the sensitivity of AML patients to antitumor drugs using pRRophetic packages. Our results indicated that the high-risk patients exhibited higher sensitivity to some potential drugs. This finding could be relevant for treatment selection and personalized therapeutic approaches in AML as it implies that high-risk patients may be more sensitive to specific antitumor drugs, which targeted to PI3K–AKT–mTOR signaling pathways. PI3K-AKT-mTOR signaling pathway is one of the most abnormal signal pathways in human cancer including AML, which is involved in the control of cell metabolism, proliferation, movement, growth and survival and many other cellular processes (38). Inhibition of PI3K-AKT-mTOR pathway is an important strategy for tumor therapy. However, the effects of these inhibitors seem to vary greatly among patients with AML (39, 40). So far, no clear mutation characteristics or other pathological processes associated with the disease have been detected to predict treatment response. Our results provide a valuable tool for individualized treatment decision-making of these drugs in AML.

It is important to acknowledge the limitations of this study. First, although we utilized large-scale datasets for the analysis, the retrospective nature of the study design may introduce inherent biases. Prospective studies are warranted to validate our findings and to assess the clinical utility of prognostic signatures and nomograms for real-time patient management. Further functional experiments and in-depth mechanistic investigations are required to elucidate the precise roles of the identified LMRGs and IRGs in AML pathogenesis and treatment responses.

In conclusion, our study presents a comprehensive analysis of the prognostic value and clinical implications of an eight-gene signature derived from LMRGs and IRGs in AML. This signature effectively stratified patients into high- and low-risk groups, demonstrating significant differences in survival outcomes and potential implications for immune cell infiltration, treatment response, and drug sensitivity. This opens up avenues for studying the interplay between lipid metabolism and immune dysregulation, which may uncover novel therapeutic targets. Future investigations could explore the manipulation of lipid metabolism pathways as a means to modulate immune responses and improve treatment outcomes in AML. Overall, these findings in this study have several broader implications. They aid in personalized risk assessment for AML patients, guiding treatment decisions towards immunotherapy or targeted drugs based on risk group assignment.

## Data availability statement

The datasets presented in this study can be found in online repositories. The names of the repository/repositories and accession number(s) can be found in the article/**Supplementary Material**.

## Ethics statement

The studies involving humans were approved by Medical Ethics Committee of The Affiliated Cancer Hospital of Zhengzhou University. The studies were conducted in accordance with the local legislation and institutional requirements. The ethics committee/institutional review board waived the requirement of written informed consent for participation from the participants or the participants’ legal guardians/next of kin because the samples used in this study were the remaining samples obtained in the course of diagnosis and treatment.

## Author contributions

DL: Conceptualization, Data curation, Funding acquisition, Investigation, Methodology, Writing – original draft, Writing – review & editing. XW: Conceptualization, Investigation, Resources, Writing – review & editing. CC: Formal analysis, Resources, Validation, Writing – review & editing. JL: Formal analysis, Methodology, Software, Visualization, Writing – review & editing. YL: Formal analysis, Validation, Writing – review & editing. HL: Formal analysis, Validation, Writing – review & editing. XG: Formal analysis, Validation, Writing – review & editing. RL: Formal analysis, Methodology, Writing – review & editing. WZ: Project administration, Resources, Supervision, Writing – review & editing. WS: Conceptualization, Investigation, Project administration, Resources, Supervision, Writing – review & editing.

## Supplementary material

Supplementary Figure 1 The relative expression of eight signature genes in the high- and low-risk groups from TCGA, GSE37642, GSE71014, and GSE12417 cohorts. **(A)** APOBEC3C, **(B)** MSMO1, **(C)** ATP13A2, **(D)** SMPDL3B, **(E)**, PLA2G4A, **(F)** TNFSF15, **(G)** IL2RA, **(H)** HGF.

Supplementary Figure 2 Correlation of each signature gene and survival in TCGA cohort. **(A)** APOBEC3C, **(B)** MSMO1, **(C)** ATP13A2, **(D)** SMPDL3B, **(E)**, PLA2G4A, **(F)** TNFSF15, **(G)** IL2RA, **(H)** HGF.

Supplementary Figure 3 The relative expression of each signature gene in the clinical samples. **(A)** APOBEC3C, **(B)** MSMO1, **(C)** ATP13A2, **(D)** SMPDL3B, **(E)** PLA2G4A, **(F)** TNFSF15, **(G)** IL2RA, **(H)** HGF.

Supplementary Figure 4 Correlation of risk score and immune checkpoints. **(A)** PD1, **(B)** PDL1, **(C)** PDL2, **(D)** CTLA4.
