# Supplementary figures and images for: A novel prognostic classification integrating lipid metabolism and immune co-related genes in acute myeloid leukemia
Source: Front Immunol. 2023 Nov 10;14:1290968. doi: 10.3389/fimmu.2023.1290968 (PMC10667441; doi:10.3389/fimmu.2023.1290968)

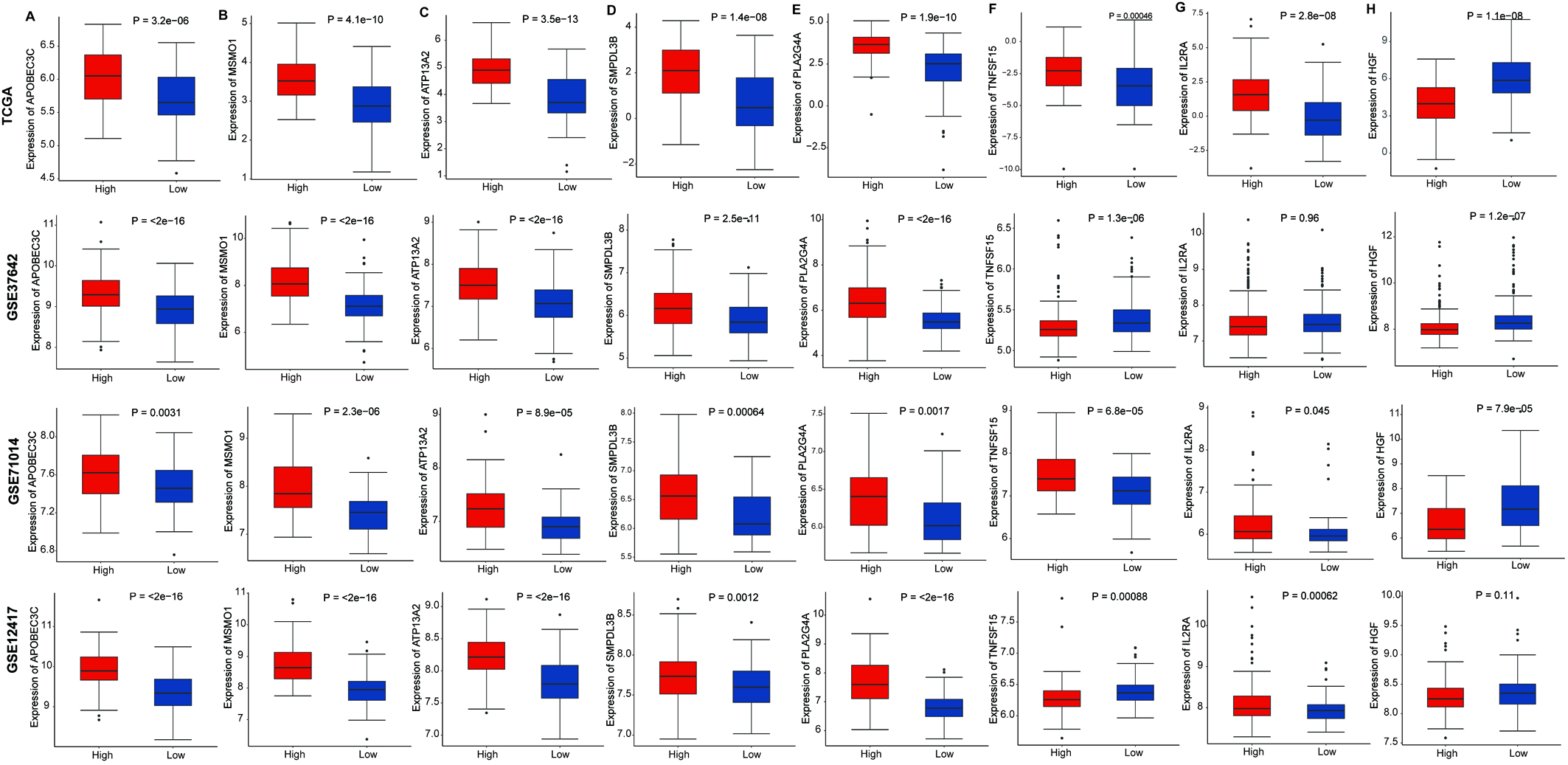

Supplement: Supplementary Figure 1 — The relative expression of eight signature genes in the high- and low-risk groups from TCGA, GSE37642, GSE71014, and GSE12417 cohorts. (A) APOBEC3C, (B) MSMO1, (C) ATP13A2, (D) SMPDL3B, (E), PLA2G4A, (F) TNFSF15, (G) IL2RA, (H) HGF. [file Image_1.tif]

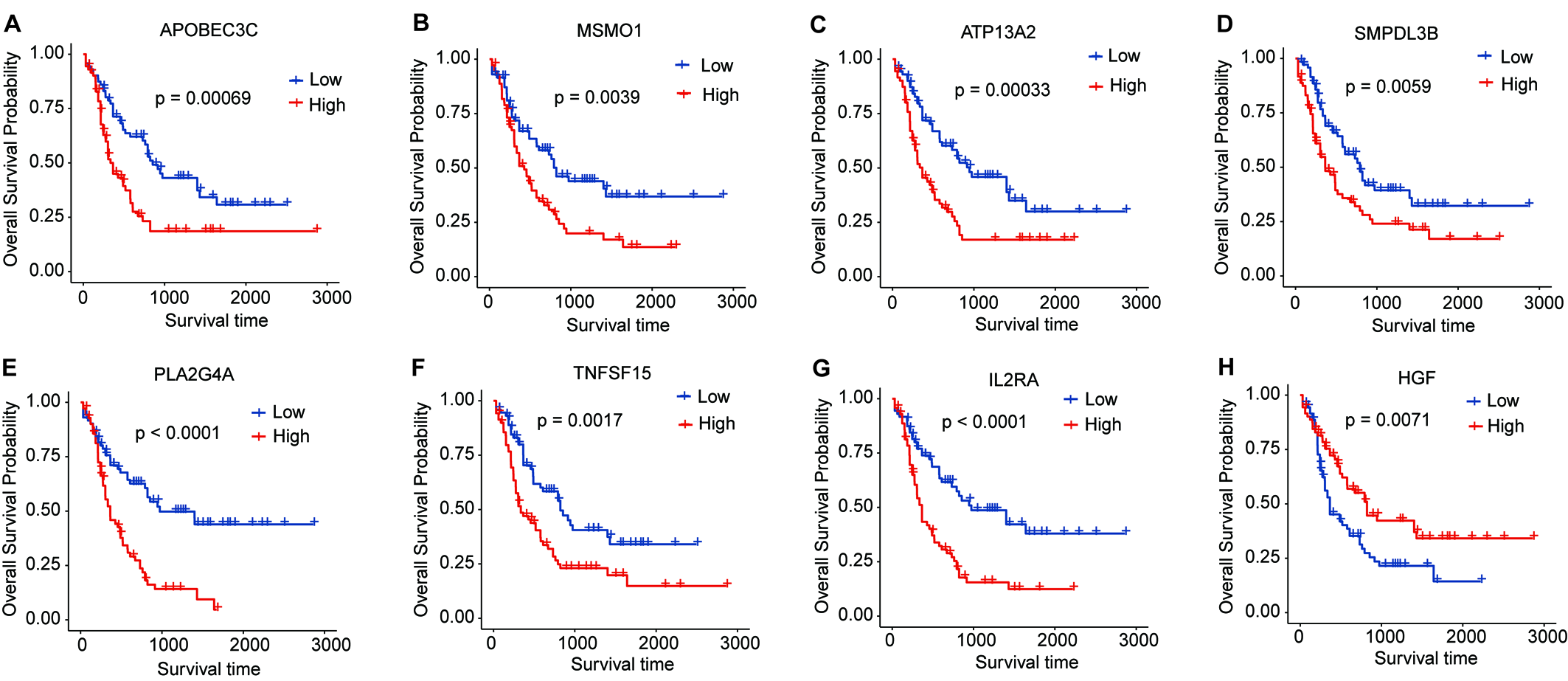

Supplement: Supplementary Figure 2 — Correlation of each signature gene and survival in TCGA cohort. (A) APOBEC3C, (B) MSMO1, (C) ATP13A2, (D) SMPDL3B, (E), PLA2G4A, (F) TNFSF15, (G) IL2RA, (H) HGF. [file Image_2.tif]

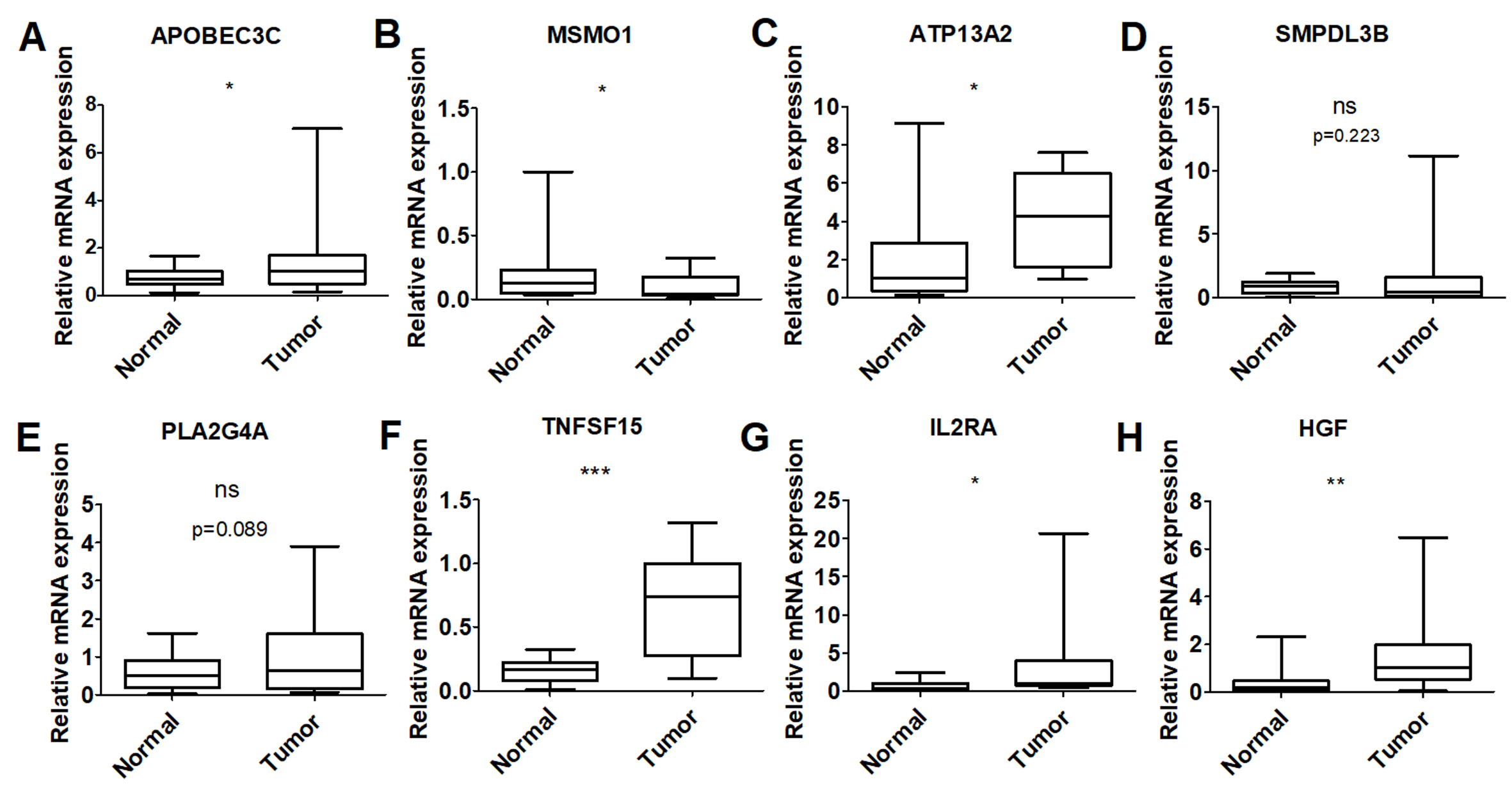

Supplement: Supplementary Figure 3 — The relative expression of each signature gene in the clinical samples. (A) APOBEC3C, (B) MSMO1, (C) ATP13A2, (D) SMPDL3B, (E) PLA2G4A, (F) TNFSF15, (G) IL2RA, (H) HGF. [file Image_3.tif]

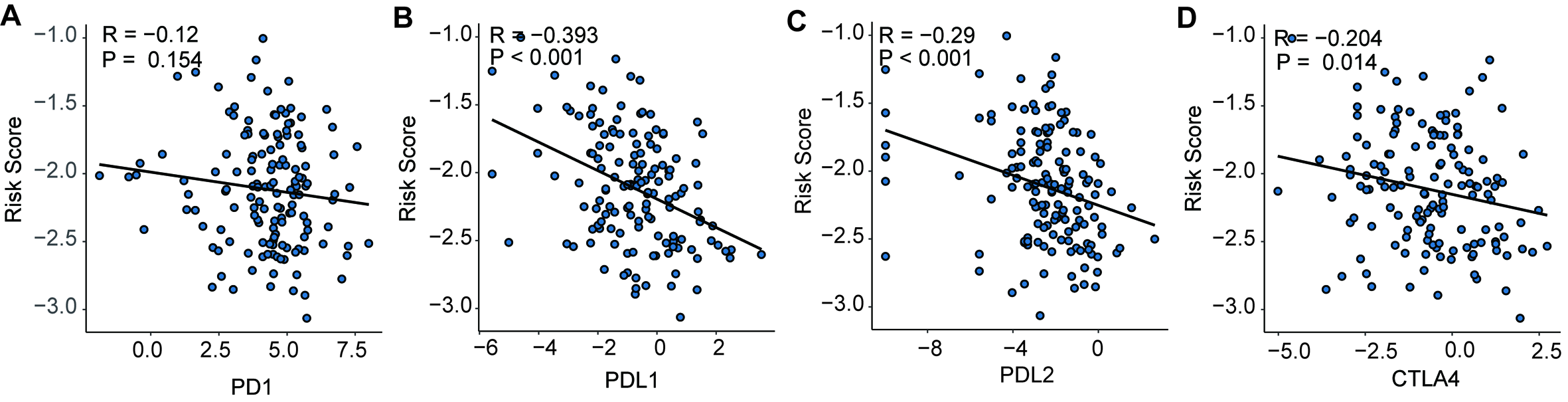

Supplement: Supplementary Figure 4 — Correlation of risk score and immune checkpoints. (A) PD1, (B) PDL1, (C) PDL2, (D) CTLA4. [file Image_4.tif]
